# Separating the effects of climate, bycatch, predation and harvesting on tītī (*Ardenna grisea*) population dynamics in New Zealand: A model-based assessment

**DOI:** 10.1371/journal.pone.0243794

**Published:** 2020-12-14

**Authors:** Sam McKechnie, David Fletcher, Jamie Newman, Corey Bragg, Peter W. Dillingham, Rosemary Clucas, Darren Scott, Sebastian Uhlmann, Phil Lyver, Andrew Gormley, Rakiura Tītī Islands Administering Body, Henrik Moller

**Affiliations:** 1 Pacific Community, Noumea, New Caledonia; 2 David Fletcher Consulting Limited, Karitane, Aotearoa New Zealand; 3 Parliamentary Commissioner for the Environment, Wellington, Aotearoa New Zealand; 4 Tokona te Ao—Tribal Economies, Te Rūnanga o Ngāi Tahu, Dunedin, Aotearoa New Zealand; 5 Department of Mathematics and Statistics, University of Otago, Dunedin, Aotearoa New Zealand; 6 Timaru, Aotearoa New Zealand; 7 Invercargill, Aotearoa New Zealand; 8 Flanders Research Institute for Agriculture, Fisheries and Food, Fisheries and Aquatic Production, Ostend, Belgium; 9 Manaaki Whenua Landcare Research, Lincoln, Aotearoa New Zealand; 10 Centre for Sustainability, University of Otago, Dunedin, Aotearoa New Zealand; MARE – Marine and Environmental Sciences Centre, PORTUGAL

## Abstract

A suite of factors may have contributed to declines in the tītī (sooty shearwater; *Ardenna grisea*) population in the New Zealand region since at least the 1960s. Recent estimation of the magnitude of most sources of non-natural mortality has presented the opportunity to quantitatively assess the relative importance of these factors. We fit a range of population dynamics models to a time-series of relative abundance data from 1976 until 2005, with the various sources of mortality being modelled at the appropriate part of the life-cycle. We present estimates of effects obtained from the best-fitting model and using model averaging. The best-fitting models explained much of the variation in the abundance index when survival and fecundity were linked to the Southern Oscillation Index, with strong decreases in adult survival, juvenile survival and fecundity being related to El Niño-Southern Oscillation (ENSO) events. Predation by introduced animals, harvesting by humans, and bycatch in fisheries also appear to have contributed to the population decline. It is envisioned that the best-fitting models will form the basis for quantitative assessments of competing management strategies. Our analysis suggests that sustainability of the New Zealand tītī population will be most influenced by climate, in particular by how climate change will affect the frequency and intensity of ENSO events in the future. Removal of the effects of both depredation by introduced predators and harvesting by humans is likely to have fewer benefits for the population than alleviating climate effects.

## Introduction

Tītī (sooty shearwater; *Ardenna grisea*) are an abundant, medium-sized petrel that breed mainly on islands around southern New Zealand and South America during the Austral summer [1]. Most of the population then migrates to the North Pacific outside the breeding season [2, 3]. They are the numerically dominant seabird species in the New Zealand region [1] and as a burrowing petrel have an important role in structuring the ecosystem on the islands on which they breed [4–6]. While tītī remain abundant, several datasets indicate that populations have declined substantially over recent decades [7–10]. A suite of mechanisms may have contributed to these declines, most of which have been widely implicated in threatening populations of other petrel species; for reviews of petrel conservation status see [11] and [12]. Sources of mortality that may have changed in recent decades, and hence could have contributed to declining populations, include depredation by introduced predators [13], bycatch in fisheries operations [14, 15], changing climatic conditions [8, 16, 17] and harvesting of chicks by humans [18].

Extinctions of tītī colonies on the New Zealand mainland have occurred in the presence of a suite of introduced predators [19, 20]. Furthermore, recent research on several islands has indicated that the introduced weka (*Gallirallus australis*), a large flightless rail native to other parts of New Zealand, and three species of introduced rat (ship rat, *Rattus rattus;* Norway rat, *R*. *norvegicus;* and Pacific rat or kiore, *R*. *exulans*) prey upon tītī eggs and young chicks [13]. As most depredation of eggs and chicks on breeding islands has been attributed to weka [21], we focus on the effect of this predator.

Bycatch in fisheries operations has also potentially impacted tītī populations, with driftnet fisheries in the North Pacific being especially culpable prior to their ban in 1991. It was estimated by [14] that between 1.0 and 12.8 million tītī were caught in fisheries operations over the period 1952–2001, and although the period of intense fishing effort coincided with declines in tītī abundance, no previous studies have attempted to estimate the demographic consequences of this mortality.

Links between large-scale climatic events and tītī abundance have previously been reported by [8] and [16]. They suggested that catch per unit effort (CPUE) of tītī chicks by human harvesters displays a lagged relationship with the Southern Oscillation Index (SOI), with years of high and low shearwater abundance pre-empting low (El Niño conditions) and high (La Niña conditions) SOI values respectively. While these studies did not investigate the demographic pathways through which SOI conditions may be affecting tītī abundance, they noted that the limited ability of the population to rebound after detrimental climatic events was consistent with adult survival, in addition to fecundity, being negatively affected during El Niño periods.

Lastly, the harvest of tītī chicks is estimated to have begun in Foveaux Strait 200–600 BP [22, 23], although scientific investigation of the harvesting system is largely restricted to the past 20 years [24]. Recent [1, 18] and historical [25] estimates of harvest offtake are now available, although the impacts of this mortality have yet to be quantified.

There are clearly many factors to consider when diagnosing the causes of population declines. Consequently, a prerequisite for optimal management of tītī populations is integrating all the available information on these mortality sources, over past decades, into a structured analysis that allows their relative influences on population growth to be assessed. No studies have yet been able to separate the relative importance of the various putative threats in influencing historical tītī population dynamics, owing to a lack of robust estimates of mortality rates and the absence of data for several key population parameters. At best, several attempts have been made to correlate indices of tītī abundance with covariates, in each case representing a single threat [8, 9]. The results are necessarily coarse and ignore the dynamics of the population underlying the abundance indices. While such observational studies have inherent problems, they are often the only choice when manipulative experiments are impractical, which is a common theme in conservation science. However, management decisions need to be made despite data deficiencies, and so it is often a matter of using the best techniques available for the given problem [26]. Using dynamical models to assess relative threats can be more appropriate than simple correlative methods, by allowing perturbations to be applied to the population at the appropriate scale (e.g. harvesting restricted to chicks or a climate covariate impacting on adult survival), with impacts affecting the population in a more realistic manner via equations representing life history. Furthermore, the likely outcomes of various competing management strategies can be assessed in a more structural manner using these models.

Therefore, in response to Rakiura Māori concerns about whether tītī will remain plentiful enough for future generations to continue birding, a study (Kia Mau Te Tītī Mo Ake Tōnu Atu–which can be translated as “Holdfast to tītī forever”) was conducted to assess harvest sustainability and advise on consequences of various management options for future birding. We develop a candidate set of population dynamics models which we fit, within a Bayesian framework, to data on the fluctuations of tītī abundance in southern New Zealand. The models are fitted to data from 1976 until 2005, the latest year for which estimates of population size and harvest rate are available. We synthesise prior information by making use of the best-available evidence on the mortality levels associated with most threatening agents [13, 14, 18, 25], demographic parameters [27, 28], and regional population size [1]. These quantities allow us to estimate the relative impacts that the major threats hypothesised by the Rakiura muttonbirding community have had on the abundance of tītī in the New Zealand region between 1976 and 2005. We use an age-structured model to determine which threats can be addressed by management actions, and at which stage of the life cycle these actions can be prescribed to achieve conservation success. Sensitivity analyses are then carried out to ensure inferences from the modelling process are robust to departures from several of the inherent assumptions. Lastly, we summarise the posterior distributions of population vital rates, which will provide the basis for future research involving population projections to predict the outcome of competing future management interventions.

## Materials and methods

### Description of the study system

The population of tītī in the New Zealand region is largely restricted to offshore islands, with particularly large numbers occurring on the Tītī Islands around Stewart Island and The Snares Islands in the New Zealand sub-Antarctic region. A detailed description of the distribution of populations in the New Zealand region was given by [1]; they also estimated the total population size to be 21.3 million individuals in the 2005 breeding season (95% CI: 19.0–23.7 million). The breeding cycle of tītī is as follows; adults return to the colony in late September–October, eggs are laid in November–early December, chicks hatch in late January–February, adults begin migrating to the Northern Hemisphere in late March and the chicks fledge and also migrate in late April–May [29].

The Tītī Islands constitute approximately 36 islands, most of which are subjected to annual traditional harvesting by Rakiura Māori (the southern-most indigenous peoples in New Zealand). Harvesting is restricted to these islands and only occurs between 1 April and 31 May each year, when pre-fledgling chicks are procured for human consumption (for detailed accounts of the harvesting system see [30]).

### Population model

We constructed an age-structured model consisting of 15 age-classes (0–14) and assumed a post-breeding census occurring immediately prior to the start of the harvesting season, which begins on the 1st April. Age-class 0 represents fully developed chicks and age-class 14 is a recycling "plus group", which accumulates all individuals older than age-class 13.

Data on individuals banded as chicks at two breeding colonies, Taiaroa Head and The Snares, were analysed by [31] in order to obtain estimates of annual juvenile survival rate, age at first return to the colony and age-specific probabilities of breeding. In modelling such data, it is necessary to assume that all individuals above a certain age have the same survival rate. Based on the analysis in [31], we therefore assumed that all individuals aged 2 years or more have the same survival rate. For simplicity we refer to this as the adult survival rate, even though some of the individuals to which it is applied are not sexually mature. In addition, such banding data cannot provide age-specific juvenile survival rates, so we assumed a single juvenile survival rate for individuals less than 2 years old.

If the population size in age-class *i* and year *t* is denoted *n*_*i*,*t*_, the model is given by

$$n_{0,t}=0.5(1-w)f_{t}N_{t}^{a}$$

$$n_{1,t}={(1-b_{t-1})s}_{t-1}^{j}(n_{0,t-1}-H_{t-1})$$

$$n_{2,t}={(1-b_{t-1})s}_{t-1}^{j}n_{1,t-1}$$

$$n_{i,t}={(1-b_{t-1})s}_{t-1}^{a}n_{i-1,t-1}\;(i=3,\ldots ,13)$$

$$\mathrm{and}\;n_{14,t}={(1-b_{t-1})s}_{t-1}^{a}(n_{13,t-1}+n_{14,t-1})$$

where *f*_*t*_ is fecundity (number of chicks alive per adult on April 1) in year *t*, $N_{t}^{a}$ is the number of adults making breeding attempts in year *t*, *w* is the probability that a breeding attempt will fail due to weka depredation, $s_{t}^{j}$ is the probability of juvenile birds alive in year *t* surviving to year *t*+1, $s_{t}^{a}$ is the probability of adult birds alive in year *t* surviving to year *t*+1, *H*_*t*_ is the number of chicks harvested by humans immediately after the census on 31 March in year *t*, and *b*_*t*_ is the proportion of birds caught as bycatch in commercial fishing operations between censuses in years *t* and *t*+1. Fecundity was multiplied by 0.5 as only one egg is laid per breeding pair.

We calculated total population size, and total number of adults, in year *t* as

$$N_{t}=\sum_{i=0}^{14}n_{i,t}\;and\;N_{t}^{a}=\sum_{i=0}^{14}p_{i}n_{i,t}$$

respectively, where *p*_*i*_ is the probability that an individual in age-class *i* is sexually mature.

Adult survival and fecundity in year *t* are assumed to be related to climatic covariates by the equations

$$s_{t}^{a}=\frac{s_{m}^{a}}{1+e^{-(\alpha_{s}+\beta_{s}z_{t}^{s})}}\;and\;f_{t}=\frac{f_{m}}{1+e^{-(\alpha_{f}+\beta_{f}z_{t}^{f})}}$$

respectively, where $s_{m}^{a}$ and *f*_*m*_ are maximum potential adult survival and fecundity, *β*_*s*_ and *β*_*f*_ are coefficients controlling the strength of the relationships with climate, and $z_{t}^{s}$ and $z_{t}^{f}$ are the values of two specific climate covariates in year *t*. We return later to the choice of $z_{t}^{s}$ and $z_{t}^{f}$. The form of these allows both positive and negative linear relationships, on the logit-scale, with the climate covariate. We used the method based on allometric relationships in [32] (see also [33]) to set a conservative maximum potential adult survival $\left(s_{m}^{a}\right)$ at 0.98. Fecundity is given by *f* = *uv*, where *u* is the probability that an adult will lay an egg and *v* is the probability that an egg will survive to become a chick on April 1. We therefore set maximum fecundity (*f*_*m*_) at 0.9, as this is just greater than the product of the maximum annual estimate of *u* (for the congeneric short-tailed shearwater, *Ardenna tenuirostris*, between 1952 and 1994) and the maximum annual estimate of *v* [28]. We assumed that survival for the first two age-classes was lower and proportional to adult survival, with $s_{t}^{j}=rs_{t}^{a}$, where *r* is a scaling parameter estimated during the modelling. This assumption of proportionality is common in population modelling of long-lived species, and it allowed juvenile survival to also have a relationship with SOI, since it implies that

$$s_{t}^{j}=\frac{{rs}_{m}^{a}}{1+e^{-(\alpha_{s}+\beta_{s}z_{t}^{s})}}.$$

The proportion of individuals caught as bycatch between years *t* and *t*+1 is *b*_*t*_ = *B*_*t*_/*N*_*t*_, where *B*_*t*_ is the total number caught between the censuses in years *t* and *t*+1, as estimated by [14], and *N*_*t*_ is the total population size in year *t*. This assumes that all individuals in the population (e.g. both breeders and non-breeders) are equally vulnerable to bycatch. We calculated the proportion of chicks that are in the population on March 31 in year *t*, and subsequently harvested by Rakiura Māori, as *h*_*t*_ = *H*_*t*_/*n*_0,*t*_.

**Fig 1:**
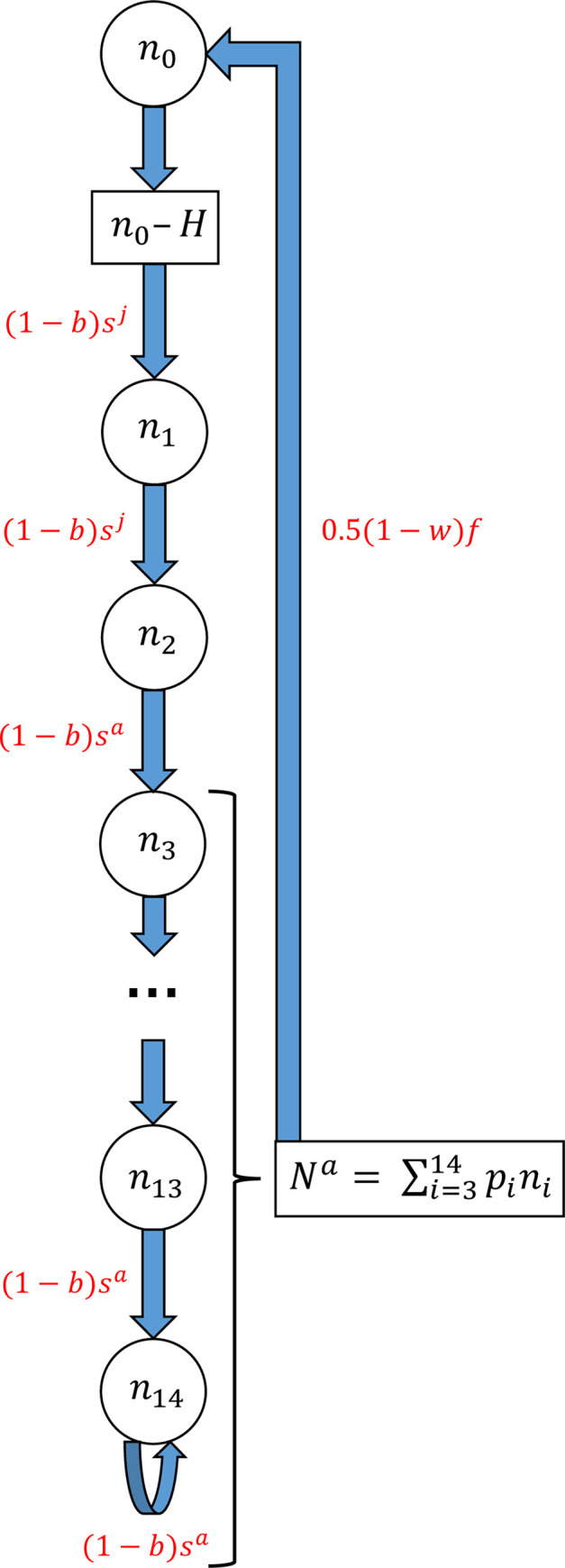
Overview of the tītī population model. The 15 age classes are represented by circles and intermediate calculations (chicks not harvested, and number of adults) by rectangles. The multipliers used to determine the number of individuals progressing to the next age-class are shown in red. The notation is as in the text, except that the dependence on year is suppressed for simplicity. Likewise, age classes 4 to 12 are suppressed for ease of presentation.

The usual method of estimating population structure in the first year of the model involves placing vague priors on population size in each age-class in that year. However, because we do not have any estimates of abundance for individual age-classes, other than for chicks in the final year (2005), this approach is not feasible. We therefore specify the initial (1976) age-structure of the population as the stable-age distribution for a Leslie matrix [34] formed by setting each demographic parameter equal to the mean of its prior distribution. As we had no prior information on the ratio of juvenile to adult survival, we set juvenile survival rate equal to the geometric mean of the 1st and 2nd year survival rates of the closely-related short-tailed shearwater [35]. We used the resulting stable-age distribution as the age-distribution in the first year of a five-year “run-in”. The length of this run-in was chosen after preliminary analyses suggested that adequate convergence occurred within that time-frame. Thus the stable age distribution from the Leslie matrix simply acted as a set of initial values used to determine an initial age distribution that was based on the demographic parameters in the model, including the model-based estimate of juvenile survival rate. The model structure during the run-in was identical to that for the actual model, except that harvesting and bycatch levels were specified as constant rates, *h* and *b*, rather than numbers of individuals. For the run-in we set *s*^*a*^, *s*^*j*^, *f*, *h*, and *b* at their 1976 values. The age-distribution in year five of the run-in was then used as the age distribution for the model proper in year 1 (1976). A similar approach has been advocated in frequentist state-space modelling [36, 37]. Fig 1 provides an overview of the population model.

### Observation model

The modelling period was restricted to 1976–2005 as no relevant data on population size or harvesting rate is available since then. Reliable records of harvest and hunting effort data were only available for the periods 1979–1992 and 1994–1998. Informed written consent was obtained from the individuals involved in the hunting, and approved by the University of Otago Ethics Committee (AEC 1–03). Although more recent harvest data are available [16], they do not provide a reliable measure of CPUE. The CPUE data we use correspond to the “Rama” period (the second half of the season), in which chicks are harvested when they emerge from burrows to fledge, as CPUE for this period is known to be proportional to chick density (R^2^ = 0.93; see [19]). The CPUE for the “Nanao” period (the first half of the season), in which chicks are harvested from the burrows, shows a much weaker relationship with chick density, and was therefore not used. CPUE was calculated by dividing the harvest tally (number of chicks) in the Rama period by the total effort that year (minutes), and then scaled to ensure that the total tally could not be identified. This scaling was part of a cultural safety agreement between the research team and the Rakiura Tītī Islands Administering Body and does not affect the modelling, as only the relative sizes of the year-to-year changes in CPUE are needed to provide the information needed on fluctuations in chick abundance. We specified the following observation model

$$I_{t}∼N({kn}_{0,t},\sigma^{2}),$$

where *I*_*t*_ is the CPUE in year *t*, *k* is the constant of proportionality, akin to the catchability coefficient used in fisheries [38], and both *k* and *σ*^2^ are estimated during the modelling.

We assumed that the observed decline in density of tītī burrows on The Snares [10] is a reliable estimate of the trend in the abundance of adult birds in the whole population, given that burrow densities are a reliable index of abundance [39], and the problems inherent in estimating trends from either beach-wrecked bird counts [9] or at-sea surveys of abundance [7]. The annual trend in adult abundance in the model is

$$\lambda_{m}={\left(\frac{N_{30}^{a}}{N_{1}^{a}}\right)}^{1/29}.$$

In order for the model to match our estimate of the trend in adult abundance, we specified that

$$\hat{\lambda }∼N(\lambda_{m},\sigma_{\lambda }^{2}),$$

where $\hat{\lambda }$ = 0.983 is the estimate of population growth rate on The Snares, and *σ*_*λ*_ = 0.002 is its standard error [10].

Adult survival rate over 1996–2004 was estimated by [27] on Whenua Hou (Codfish Island) and The Snares. In order for the model to match the estimate for this period, we specified that

$${\hat{l}}^{a}=\log\left\{{\hat{s}}^{a}/(s_{m}^{a}-{\hat{s}}^{a})\right\}∼N({\bar{l}}^{a},\sigma_{a}^{2})$$

where

$${\bar{l}}^{a}=\frac{1}{9}\sum_{t=1996}^{2004}\log\{s_{t}^{a}/(s_{m}^{a}{-s}_{t}^{a})\},$$

${\hat{s}}^{a}$ = 0.952 is the estimate of annual adult survival rate, ${\hat{l}}^{a}$ = 2.987 is the corresponding estimate of the logit of adult survival rate and *σ*_*a*_ = 0.422 is the standard error of ${\hat{l}}^{a}$. Adult survival is likely to be related to broad-scale factors, and should therefore be similar on each island. The value of ${\hat{s}}^{a}$ is therefore the mean estimate for Whenua Hou and The Snares [27].

Fecundity was estimated by [28] for the periods 1997−1999 and 2003−2005. In order for the model to match the estimate for this period, we specified that

$${\hat{l}}^{f}=\log\left\{\hat{f}/(f_{m}-\hat{f})\right\}∼N({\bar{l}}^{f},\sigma_{f}^{2})$$

where

$${\bar{l}}^{f}=\frac{1}{6}\left\{\sum_{t=1997}^{1999}\log\left\{f_{t}/(f_{m}-f_{t})\right\}+\sum_{t=2003}^{2005}\log\left\{f_{t}/(f_{m}-f_{t})\right\}\right\},$$

$\hat{f}$ = 0.52 is the estimate of fecundity, ${\hat{l}}^{f}$ = 0.08 is the corresponding estimate of the logit of fecundity and *σ*_*f*_ = 0.108 is the standard error of ${\hat{l}}^{f}$. Estimates of the probability that a sexually mature individual will lay an egg (*u*) do not exist for tītī, so we used an estimate available for the congeneric short-tailed shearwater as a prior for this parameter (Stuart Bradley, unpublished data). The probability that an egg laid will survive to become a chick (*v*) has been estimated during long-term monitoring at The Snares and Whenua Hou [28]. It is difficult to assess how the value of this parameter for the whole population would relate to estimates from individual islands, as this vital rate is likely to be more site-specific than survival, owing to potential differences in rainfall, burrow quality, etc. between islands. We used a precision-weighted mean rate over the two islands to obtain $\hat{v}$ and hence $\hat{f}=\hat{u}\hat{v}$.

The mark-recapture models used by [31] to estimate juvenile survival rate, at both Taiaroa Head and The Snares during the period 1996–2003, cannot distinguish juveniles that die from those that do not return to their natal colony, which will lead to underestimation of the true juvenile survival rate. We therefore ensured that the mean (on the logistic scale) of the model-based juvenile survival rate over this period was at least as big as the larger of these two estimates (Taiaroa Head), by specifying that

$${\hat{l}}^{j}=\log\left\{{\hat{s}}^{j}/(1-{\hat{s}}^{j})\right\}∼N({r_{j}\bar{l}}^{j},\sigma_{j}^{2})$$

where

$${\bar{l}}^{j}=\frac{1}{8}\sum_{t=1996}^{2003}\log\{s_{t}^{j}/(1{-s}_{t}^{j})\},$$

${\hat{s}}^{j}$ = 0.566 is the estimate of annual juvenile survival rate from Taiaroa Head, ${\hat{l}}^{j}$ = 0.266 is the corresponding estimate of the logit of juvenile survival rate, *σ*_*j*_ = 0.155 is the standard error of ${\hat{l}}^{j}$, and *r*_*j*_ is a parameter between 0 and 1 that is estimated during the modelling.

Finally, an independent study estimated the number of tītī in age-class 0 in the New Zealand region in 2005 to be ${\hat{n}}_{0,30}$ = 2.787 million [1]. In order for the model to match this estimate, we therefore specified that

$${\hat{n}}_{0,30}∼N(n_{0,30},\sigma_{n}^{2}),$$

where *σ*_*n*_ = 0.132 is the standard error of ${\hat{n}}_{0,30}$ [1].

### Input variables

We used estimates of bycatch of tītī over the period 1952–2001 [14] to set the values for *B*_*t*_ in those years. While some bycatch still occurred during the period 2002–2005, in both active and passive fishing gears [15, 40–42], the magnitude is believed to be low relative to the period before driftnet fisheries were banned in 1991. In addition, the estimate of total tītī bycatch in New Zealand fisheries has been less than 1200 in each fishing season between 2002–03 and 2016–2017 [43], which amounts to less than 0.01% of the estimate of the New Zealand adult population size (12.8 million) for the period 1994–2005 [1]. We therefore assumed bycatch was negligible during the period 2002–2005, and set the corresponding values of *B*_*t*_ to zero. We used estimates of chick harvest during 1976–2005 [25] to set the corresponding values of *H*_*t*_. Sensitivity analyses were carried out in order to investigate the consequences of bias in the assumed harvest and bycatch levels.

Most depredation of eggs and chicks on breeding islands has been attributed to weka [22]. Norway rats probably also affect productivity, although this has yet to be quantified [13]. In addition, Norway rats are only present on a few of the smaller islands where tītī breed, and for the purposes of modelling the entire New Zealand population their impacts are likely to be insignificant, so we do not estimate their effects. We calculated a regional rate of depredation of chicks by weka (11%) by multiplying the rate estimated by [22] by the proportion of the New Zealand population estimated to be present on islands where weka existed in 2005 [1]. This rate was used as the value of *w* in the population model.

### Priors

We use an informative prior for the probability of being sexually mature in age-class *i* (*p*_*i*_), based on the results in [31]. This probability was set to zero for age-classes 0–2, and to 1 for age-classes 13 and 14. For age classes 3 to 12, it was assumed to be a linear function of age on the probit scale, i.e.

$$p_{i}=\Phi \left(\frac{i-\mu_{p}}{\sigma_{p}}\right)$$

where *Φ*(.) is the cumulative distribution function for the standard normal distribution. The parameters *μ*_*p*_ and *σ*_*p*_ are the mean and standard deviation of age at maturity, which were estimated by [31] to be 7.5 years and 1.9 years respectively. We specified the prior for (*μ*_*p*_, *σ*_*p*_) as a bivariate normal distribution with mean (7.5,1.9) and variance-covariance matrix as estimated by [31]. We also used the following independent vague priors [44]: we gave both *β*_*s*_ and *β*_*f*_ a normal prior with mean 0 and standard deviation 100, both *k* and *σ* had a uniform prior on [0,1000], *r* and *r*_*j*_ both had a uniform prior on [0,1], and we gave $N_{1}^{a}$ a uniform prior on [0,100] (million adults).

### Selection and formulation of climate indices

We used results from the previous investigation of the relationship between climate and CPUE of harvesters by [8] to formulate climate indices. This led to using the monthly Troup SOI [45], which measures the standardised anomaly of the mean sea level pressure difference between Tahiti and Darwin. We obtained the monthly Troup index, with a base period of 1951−2000, from Brett Mullan at the National Institute of Water and Atmospheric Research in Wellington, New Zealand. Large negative and positive SOI values indicate strong El Niño and La Niña events, respectively. As survival and fecundity operate over different time-periods, we calculated different annual SOI measures for adult survival and fecundity in year *t*, which we denote as $z_{t}^{s}$ and $z_{t}^{f}$ respectively. We defined $z_{t}^{s}$ to be the mean of the 12 monthly SOI values between April in year *t* to the following March, as this covers the period that survival is calculated over. Likewise $z_{t}^{f}$ is the 12-month mean between October (just before the onset of egg-laying) in year *t*−1 and the following September. We investigated the possibility of a lagged relationship between vital rates and climate indices (e.g. [8]). Thus we define an S0 model to be one with a “zero lag” on adult survival and an F0 model to be one with a zero lag on fecundity, i.e. the equations relating these two rates to SOI are exactly as described above. In contrast, an S–1 model is defined to be one with a –1 lag on survival, the function involving $z_{t}^{s}$ now being a model for $s_{t-1}^{a}$, i.e.

$$s_{t-1}^{a}=\frac{s_{m}^{a}}{1+e^{-(\alpha_{s}+\beta_{s}z_{t}^{s})}}.$$

Likewise, an S1 model has a +1 lag on survival, with

$$s_{t+1}^{a}=\frac{s_{m}^{a}}{1+e^{-(\alpha_{s}+\beta_{s}z_{t}^{s})}}.$$

Analogous definitions hold for an F–1 model and an F1 model. A model with 0-lags on both adult survival and fecundity is denoted S0F0, with similar definitions for other lags. Based on the optimal lags identified by [8], we considered models involving all combinations of lags −1, 0 and +1, for both and adult survival and fecundity, resulting in nine models. To ensure that we were identifying the optimal lags of the climate indices, we monitored whether the fit of any of the models was highest at the boundaries of the set of lags we were investigating. For instance, if the fit was best for a model with the most extreme lag in the candidate set, we formulated new models incorporating climate indices with more extreme lags, until the fit became worse. S1 Table gives the values of $z_{t}^{f}$ and $z_{t}^{s}$ that were used in the modelling, and Table 1 provides an overview of how the data inform the model.

**Table 1:** Overview of how the data inform the tītī population model.

Estimates and input variables	Population model parameters informed
Estimate (with SE) of chick abundance in 2005 (${\hat{n}}_{0,30}$ and *σ*_*n*_)	Chick abundance in 2005 (*n*_0,30_)
Estimate of trend in burrow density 1976–2005 ($\hat{\lambda }$ and *σ*_*λ*_)	Trend in adult abundance 1976–2005 ${(N}_{30}^{a}/N_{1}^{a})$
CPUE in 1979–92, 1994–98 (*I*_*t*_ for *t* = 4,…,17,19,…,23)	Chick abundance in 1979–92, 1994–98 (*n*_0,*t*_ for *t* = 4,…,17,19,…,23)
Overall adult survival (with SE) in 1996–2004 (${\hat{s}}^{a}$ *and σ*_*a*_)	Adult survival in 1996–2004 ($s_{t}^{a}$ for *t* = 21,…,29)
Overall juvenile survival (with SE) in 1996–2003 (${\hat{s}}^{j}$ *and σ*_*j*_)	Juvenile survival in 1996–2003 ($s_{t}^{j}$ for *t* = 21,…,28)
Fecundity (with SE) in 1997–99, 2003–05 ($\hat{f}$ and *σ*_*f*_)	Fecundity in 1997–99, 2003–05 98 (*f*_*t*_ for *t* = 22,…,24,28,…,30)
Age at sexual maturity (with covariance matrix) (*μ*_*p*_ and *σ*_*p*_)	Age at sexual maturity (*p*_*i*_ for *i* = 0,…,14)
Bycatch total in 1976–2004 (*B*_*t*_ for *t* = 1,…,29)	Bycatch rate in 1976–2004 (*b*_*t*_ for *t* = 1,…,29)
Chick harvest in 1976–2004 (*H*_*t*_ for *t* = 1,…,29)	Chick harvest in 1976–2004 (*H*_*t*_ for *t* = 1,…,29)
Chick depredation by weka (*w*)	Chick depredation by weka (*w*)
Mean (Oct-Sep) SOI during 1974–2005 ($z_{t}^{f}$ *for* t = −1,…,30)	Fecundity in 1976–2005 (*f*_*t*_ for *t* = 1,…,30)
Mean (Apr-Mar) SOI during 1975–2006 ($z_{t}^{s}$ *for* t = 0,…,31)	Adult and juvenile survival in 1976–2005 ($s_{t}^{a}$ and $s_{t}^{j}$ for *t* = 1,…,29)
Maximum adult survival ($s_{m}^{a}$)	Adult survival in 1976–2005 ($s_{t}^{a}$ for *t* = 21,…,29)
Maximum fecundity (*f*_*m*_)	Fecundity in 1976–2005 (*f*_*t*_ for *t* = 1,…,30)

### Modelling procedures, sensitivity analyses and model fit

Our modelling procedure comprised two steps; selecting appropriate climate indices and, given the selected indices, investigating the sensitivity of our inferences to both changes in input values and alternative datasets. We compared the fit of models by calculating the Watanabe-Akaike Information Criterion (WAIC; [46]). We summarised the WAIC-values by calculating the WAIC-based model weight *w*_*m*_ for model *m* as

$$w_{m}=\frac{e^{-0.5\Delta W_{m}}}{\sum_{k=1}^{M}e^{-0.5\Delta W_{k}}}$$

where *ΔW*_*m*_ = *W*_*m*_−min(*W*_*m*_), *W*_*m*_ is the WAIC-value for model *m* and *M* is the total number of models [47]. We compared the best model (i.e. with the highest WAIC weight) with one in which only adult survival was related to climate (with the same lag in the best model), and fecundity was assumed to be constant each year (denoted NoF). We repeated this for a model where only fecundity was related to climate (with the same lag as in the best model) and adult survival was assumed to be constant each year (denoted NoS). For completeness, we also included a model with both adult survival and fecundity assumed to be constant each year (NoSNoF). We also used model averaging, based on the WAIC weights, in order to allow for model uncertainty when calculating posterior means and 95% credible intervals [48].

Ideally analyses of population time-series using demographic models include both observation and process error. State-space models provide a means to fit such models within a Bayesian or frequentist framework, and have received substantial interest in population ecology [49–52]. Unfortunately, many time-series contain insufficient information to achieve identifiability of all parameters in these models, especially variance terms [53]. This is true for our situation, where only part of the population is observable, and so, in the absence of prior information on the absolute (or relative) magnitude of process and observation error, our attempts to incorporate process error into the models were unsuccessful, with the MCMC sampler unable to converge on a stationary distribution. We therefore used an observation error-only model. When state-space modelling is prohibitive, this appears to be preferable to process error-only models [54, 55]; the latter often result in severely biased parameter estimates [56], which are largely avoided in observation error-only models [55]. In addition, there is evidence that fitting complex models that are not supported by the data can have severe consequences for management advice [57]. Having said that, we would expect parameter estimates from an observation-error-only model to be overly precise. We therefore carried out a sensitivity analysis, in order to gauge the extent to which our models might be overly optimistic about the precision with which we can estimate the parameters. We modified the best-fitting model by adding process error to the relationship between SOI and both adult survival and fecundity, with pre-specified “high” values for the two error standard deviations. In order to determine these high values, we specified a minimum and maximum value for the true annual adult survival and fecundity rates for the period 1976–2005. We then calculated the process-error standard deviations that would lead to the modified model predicting that the true adult survival and fecundity rates for this period cover the range specified by the minimum and maximum. We then fitted the modified best model and assessed the extent to which the results differed from those given by the original best model, in terms of the relationships between SOI and both adult survival and fecundity, the posterior prediction intervals for CPUE, and the estimated effects of climate, harvest, bycatch, and weka depredation during the period 1976–2005.

A second type of sensitivity analysis involved identifying input parameters and prior values which were difficult to specify during formulation of the model, and assessing the sensitivity of our results to these. For example, it is difficult to obtain estimates of fecundity $\left(\hat{f}\right)$ and population growth $\left(\hat{\lambda }\right)$ for the entire New Zealand population, as monitoring was restricted to a subset of individual islands. We fitted 11 alternative models, all being variations of the best-fitting model (Table 2), and monitored the differences in key model outputs. Only one component of the best-fitting model was changed in each alternative model, as the computational burden of investigating interactions between components was deemed to not be feasible.

**Table 2:** Description of alternatives to the best model (S-1F0) that were fitted in the sensitivity analysis.

Model	Parameter	Original	New	Description
**B-HIGH1**	*B*_*t*_	Estimate	Upper limit 1	Bycatch at upper limit of Uhlmann et al. (2005)
**B-HIGH2**	*B*_*t*_	Estimate	Upper limit 2	Bycatch at upper limit of Uhlmann et al. (2005) with *B*_*t*_ for 1991–2005 at estimate in 1990 (before driftnet ban)
**F-LOW**	$\hat{f}$	0.520	0.416	Mean observed fecundity = 0.8 x estimate
**F-HIGH**	$\hat{f}$	0.520	0.624	Mean observed fecundity = 1.2 x estimate
**FM-LOW**	*f*_*m*_	0.9	0.8	Maximum fecundity rate = 0.8
**SM-HIGH**	$s_{m}^{a}$	0.980	0.999	Maximum survival rate = 0.999
**H-LOW**	*H*_*t*_	Estimate	Lower limit	Harvest at lower limit of Bragg et al. (2007)
**H-HIGH**	*H*_*t*_	Estimate	Upper limit	Harvest at upper limit of Bragg et al. (2007)
**T-LOW**	$\hat{\lambda }$	0.983	0.973	Observed trend one percentage-point below estimate
**T-HIGH**	$\hat{\lambda }$	0.983	0.993	Observed trend one percentage-point above estimate

We used posterior predictive checking [44] to assess the adequacy of the best model in estimating CPUE. This involved comparing the observed CPUE with the model-averaged posterior predictive distribution of CPUE, this distribution being summarised by a 95% prediction interval.

We fitted the models using the package “rjags” in R version 3.6.2 [58], which uses the software JAGS (http://mcmc-jags.sourceforge.net/; version 4.3.0) to implement Markov Chain Monte Carlo (MCMC) methods to sample from the posterior distributions of the parameters. We ran two chains for 200,000 iterations, discarding the first 100,000 as a “burn-in”, and thinned the chains to 1 in 20, resulting in 5,000 samples from each chain being used for inference. Convergence of the sampler was assessed by the Brooks-Gelman-Rubin potential scale reduction factor ${\hat{R}}_{c}$ [59] and by visual inspection of trace plots. Estimation of parameters is summarised by posterior means and 95% central credible intervals, unless otherwise stated.

## Results

The model that best fitted the data was S–1F0, with a WAIC weight of 0.49 (Table 3). This model includes a relationship between adult survival and SOI lagged by one year, and no lag between fecundity and SOI (Table 3). The next best model (S–2F0) had a much smaller weight of 0.21. All of the best five models (total weight = 0.95) include a relationship between fecundity and SOI without a lag, and all of the top four models (total weight = 0.91) include a relationship between adult survival and SOI. Models that included only one, or neither, of the relationships between climate and the two vital rates were given little weight (Table 3). All of the results from these models are based on model averaging using the WAIC weights, except when the parameter of interest does not have the same interpretation in all models, as model averaging is then inappropriate [46]; in such a case, we present results from the best model (S–1F0).

**Table 3:** ΔW values and WAIC model weights for models with different relationships between adult survival and SOI, and fecundity and SOI.

Model	ΔW	Weight
**S**–**1F0**	0.0	0.49
**S**–**2F0**	1.7	0.21
**S1F0**	2.0	0.17
**S0F0**	4.6	0.04
**NoSF0**	4.7	0.04
**S**–**1NoF**	6.0	0.02
**S**–**1F**–**1**	6.8	0.02
**S**–**1F1**	8.2	0.01
**S0F**–**1**	13.4	0.00
**NoSNoF**	15.2	0.00
**S0F1**	18.0	0.00
**S1F**–**1**	18.7	0.00
**S1F1**	19.5	0.00

**Fig 2:**
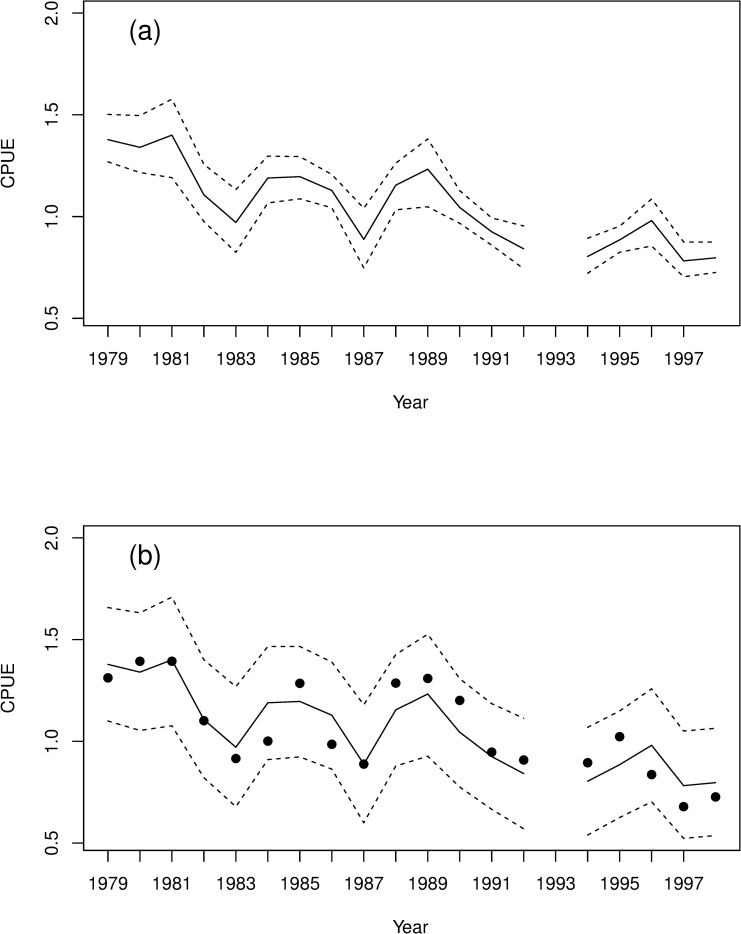
Fit of the demographic models to harvest data. Observed and predicted catch per unit effort (CPUE) data from harvesting of tītī chicks in New Zealand during the period 1979–1998. In both plots the solid line shows the model-averaged posterior mean for CPUE. In (a) the dashed lines show the 95% model-averaged credible interval; in (b) the dots are the observed CPUE values and the dashed lines show the 95% model-averaged 95% prediction interval. Model averaging was performed using WAIC model weights.

The model-averaged predictions appear to provide an adequate description of the data (Fig 2), capturing the fluctuations in abundance with a good level of precision (Fig 2A), and each of the observed CPUE values lies inside the corresponding 95% model-averaged prediction interval (Fig 2B).

**Fig 3:**
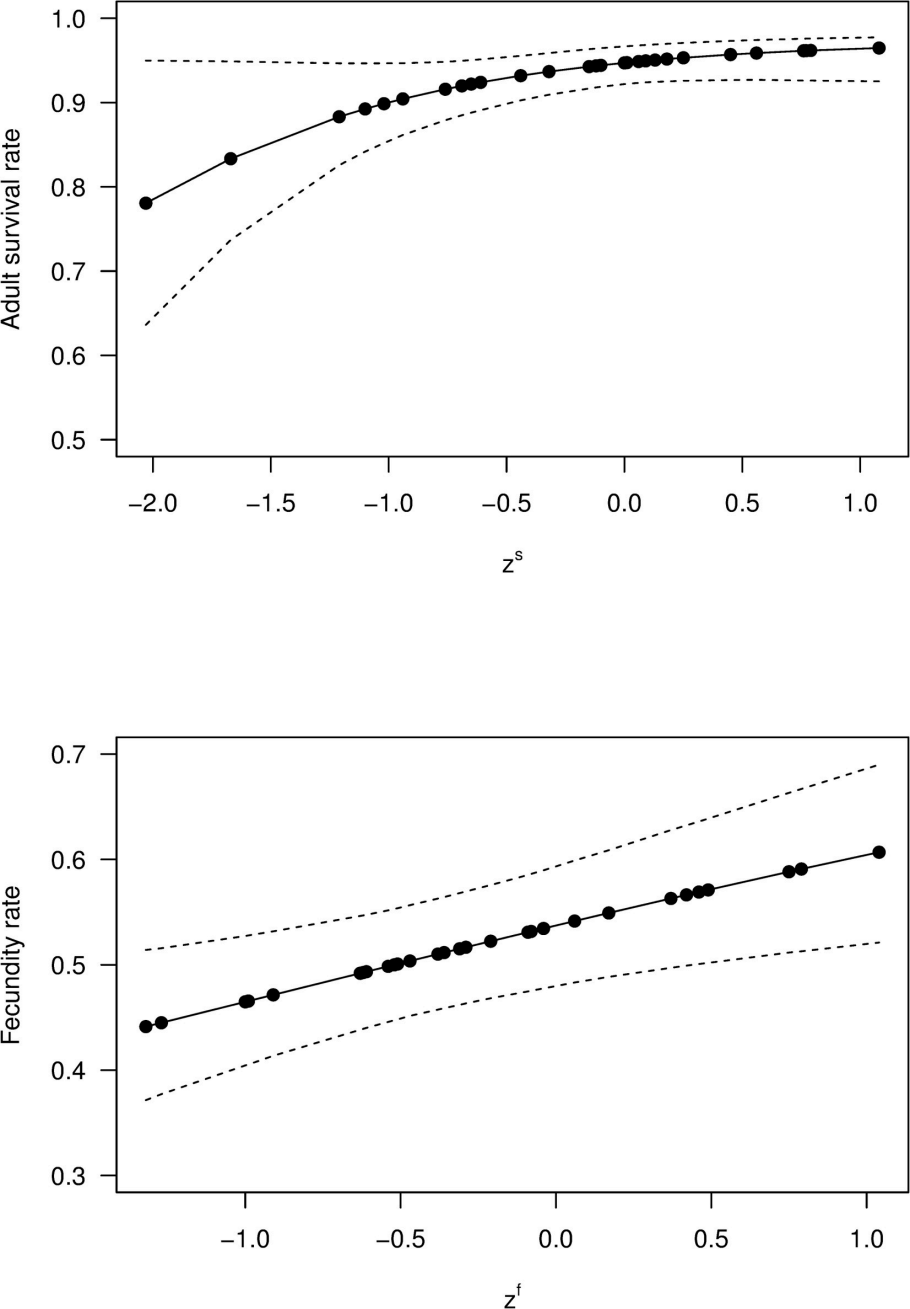
Southern Oscillation Index versus adult survival and fecundity of tītī. Estimated relationship, based on the best model (S–1F0), between SOI and both adult survival rate and fecundity rate, for tītī in New Zealand during the period 1976–2005, plotted against the observed values of $z_{t}^{s}$ and $z_{t}^{f}$ respectively (dots), together with 95% credible intervals (dashed).

Parameter estimates from the best-fitting model (S–1F0) revealed clear relationships between vital rates and SOI, with the posterior probabilities that *β*_*f*_ > 0 and *β*_*s*_ > 0 being 0.99 and 0.97 respectively. The estimated relationship between adult survival and SOI showed a pronounced reduction in survival for very low SOI values, although the occurrence of such values in the data was rare (Fig 3). The model estimated that fecundity increases in a roughly linear manner with increasing SOI (Fig 3), the predicted reduction in mean fecundity between the years with the highest and lowest observed SOI values being approximately 0.17. These results imply that adult survival will generally be lower (higher) in the year before an El Niño (La Niña), while fecundity will be lower (higher) in the year of an El Niño (La Niña). The other two models with a WAIC weight greater than 0.1 (S–2F0 and S1F0) had the same type of positive relationship between SOI and both adult survival and fecundity, the main differences being that these models had slightly higher values for *β*_*f*_ and smaller values for *β*_*s*_, compared to the best model.

**Fig 4:**
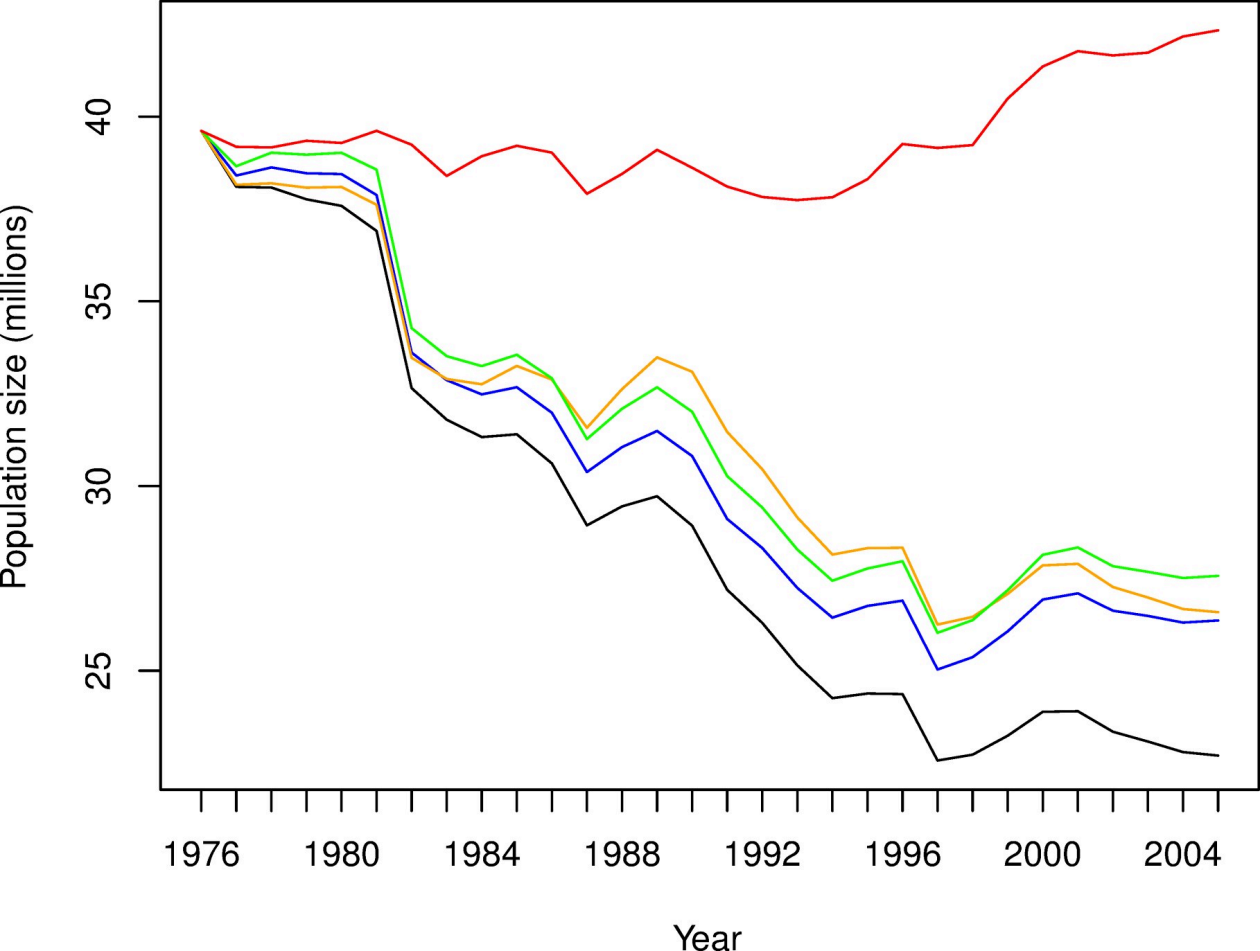
Effects of climate, harvesting, bycatch, and weka-depredation on tītī population dynamics. Model-averaged posterior mean of population size of tītī in the New Zealand region for the period 1976–2005 (black), together with the model-averaged posterior mean of the predicted population size in the absence of harvest (blue), absence of bycatch (orange), absence of weka (green), and absence of a relationship between SOI and adult survival (red). Model averaging was performed using WAIC model weights.

The model-averaged posterior mean for population size shows a steady decline over the period 1976–2005, interspersed with several seasons of abrupt declines (Fig 4; black line). The largest decline occurred in 1982, with a strong, sustained period of decline in the late 1980s and early 1990s, when a series of negative SOI values occurred. The population is predicted to have declined from 39.6 million in 1976 to 22.7 million in 2005, a decline of 1.9% (1.5%–2.3%) per year (Fig 4). As we would expect, this is close to the estimated decline of 1.7% per year that was used to fit the model. We now consider the historical impact of those threats that are open to management; harvesting, bycatch and weka depredation. In doing so we generated three independent “virtual populations”, in which there was no harvest, no bycatch, or no depredation by weka (Fig 4; blue, orange and green lines respectively). These impacts were estimated to be similar, with the model-averaged posterior mean for the decline over the period 1976–2005 being 1.4% (0.9–1.9%), 1.4% (0.9–1.8%) or 1.2% (0.7–1.8%) for a population not exposed to harvest, bycatch or weka, respectively. The impact of the relationship between SOI and adult survival is stronger than that from any of the threats that are directly open to management (Fig 4; red line), with the model-averaged posterior mean for a population not exposed to this climate-related threat being relatively stable, with an annual growth rate of 0.01%. However, the uncertainty around this projection was high, with the 95% credible interval lying between an annual decline of 2.2% and an annual increase of +2.4%. The impact of the relationship between SOI and fecundity is not shown in Fig 4, as the effect was negligible. This is not surprising, given the fact that population growth rate of a long-live species is known to be far less sensitive to fecundity than to adult survival.

The proportion of chicks in the total population that were estimated to be harvested was relatively low, the model-averaged posterior mean for the mean proportion over all years being 0.11 (0.09–0.12), with that for the minimum and maximum over all years being 0.07 (0.06–0.08) and 0.15 (0.13–0.18) respectively. Bycatch of tītī in fisheries operations initially increased from the start of the time-series and reached a peak around the mid-late 1980s, before reducing dramatically after the ban of North Pacific drift nets in 1992. The model-averaged posterior mean for the maximum bycatch mortality rate over all years is 0.016 (0.013–0.020).

The first sensitivity analysis involved modifying the best-fitting model (S–1F0) to include process errors in adult survival and fecundity, i.e. we set

$$s_{t-1}^{a}=\frac{s_{m}^{a}}{1+e^{-(\alpha_{s}+\beta_{s}z_{t}^{s}+\varepsilon_{t}^{s})}}\;and\;f_{t}=\frac{f_{m}}{1+e^{-(\alpha_{f}+\beta_{f}z_{t}^{f}+\varepsilon_{t}^{f})}}$$

where $\varepsilon_{t}^{s}$ and $\varepsilon_{t}^{f}$ are the process errors, with $\varepsilon_{t}^{s}∼N(0,\sigma_{s}^{2})$ and $\varepsilon_{t}^{f}∼N(0,\sigma_{f}^{2})$. We chose a high value for *σ*_*s*_ by specifying that this new model would predict that the true adult survival rates during 1976–2005 cover the range 0.65 to 0.97, with 0.65 representing a very low adult survival rate for a petrel species and 0.97 being just below the specified maximum ($s_{m}^{a}$). We did so by setting

$$\frac{s_{m}^{a}}{1+e^{-(\alpha_{s}+\beta_{s}z_{min}^{s}–2\sigma_{s})}}=0.65\;and\;\frac{s_{m}^{a}}{1+e^{-(\alpha_{s}+\beta_{s}z_{max}^{s}+2\sigma_{s})}}=0.97,$$

where $z_{min}^{s}$ = –2.03 and $z_{max}^{s}$ = 1.08 are the minimum and maximum value of $z_{t}^{s}$ observed during the period 1976–2005 (Fig 3), and *α*_*s*_ and *β*_*s*_ are set at their posteriors means, as given by the original S–1F0 model. Solving these two equations gives *σ*_*s*_ = 0.37 and *σ*_*s*_ = 0.10 respectively, so we set *σ*_*s*_ at the maximum of these two values. Likewise, we chose a high value for *σ*_*f*_ by specifying that the modified model would predict that the true fecundity rates during 1976–2005 cover the range 0.2 to 0.8 (approximately 60% lower and 60% higher respectively than the estimate of 0.52 in [28]), and therefore set

$$\frac{f_{m}}{1+e^{-(\alpha_{f}+\beta_{f}z_{min}^{f}–2\sigma_{f})}}=0.2\;and\;\frac{f_{m}}{1+e^{-(\alpha_{f}+\beta_{f}z_{max}^{f}+2\sigma_{f})}}=0.8,$$

where $z_{min}^{f}$ = –1.32 and $z_{max}^{f}$ = 1.04 are the minimum and maximum value of $z_{t}^{f}$ observed during the period 1976–2005 (Fig 3), and *α*_*f*_ and *β*_*f*_ are set at their posteriors means, as given by the original S–1F0 model. Solving these two equations gives *σ*_*f*_ = 0.56 and *σ*_*f*_ = 0.72 respectively, so we set *σ*_*f*_ at the maximum of these two values.

**Fig 5:**
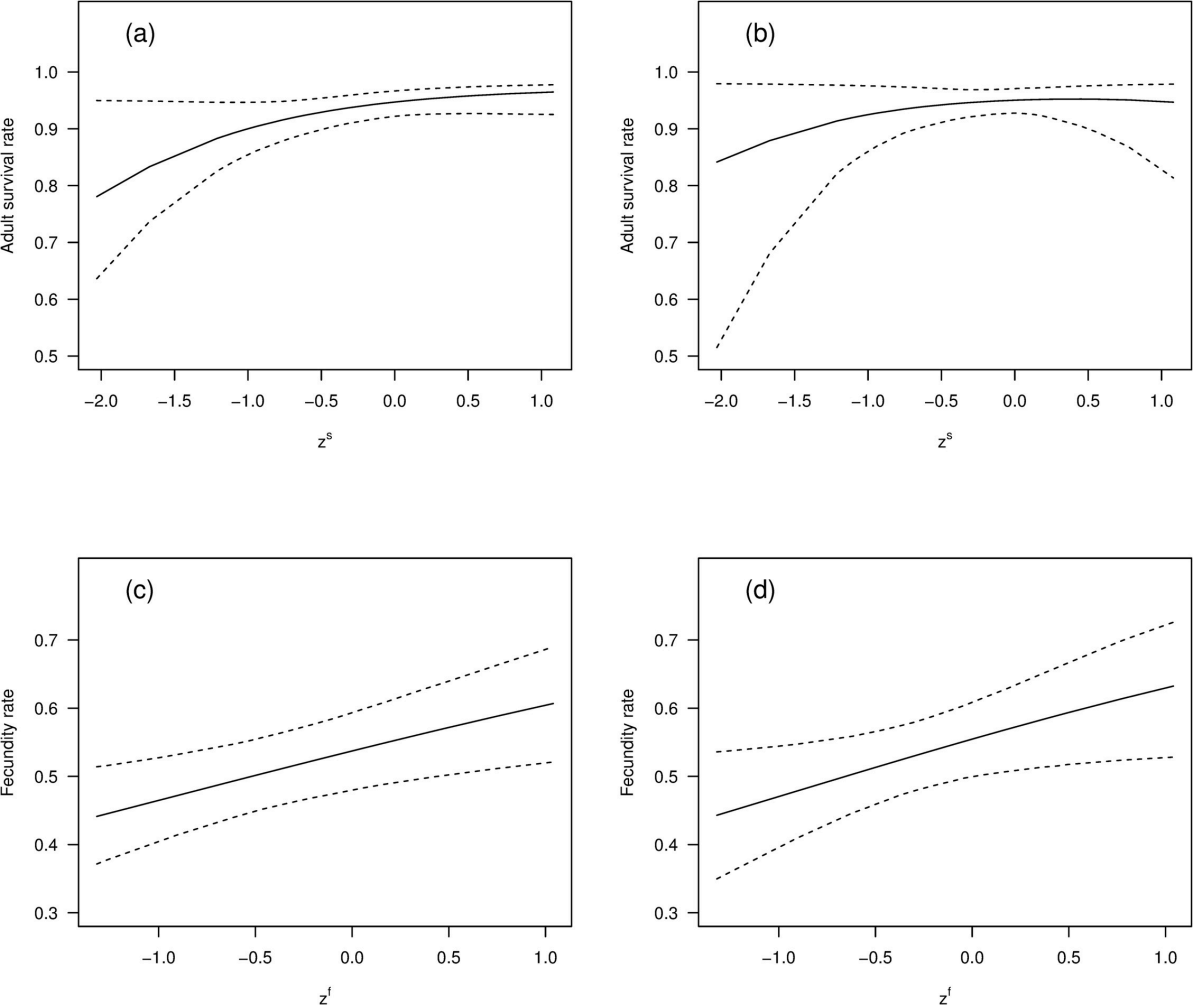
Effects of including process error in the best-fitting model on the relationship between SOI and both adult survival and fecundity. Estimated relationships (solid) between a) adult survival from the best model (S–1F0) and $z_{t}^{s}$; b) adult survival from the modified best model and $z_{t}^{s}$; c) fecundity from the best model (S–1F0) and $z_{t}^{f}$; d) fecundity the modified best model and $z_{t}^{f}$. The dashed lines are 95% credible intervals.

**Fig 6:**
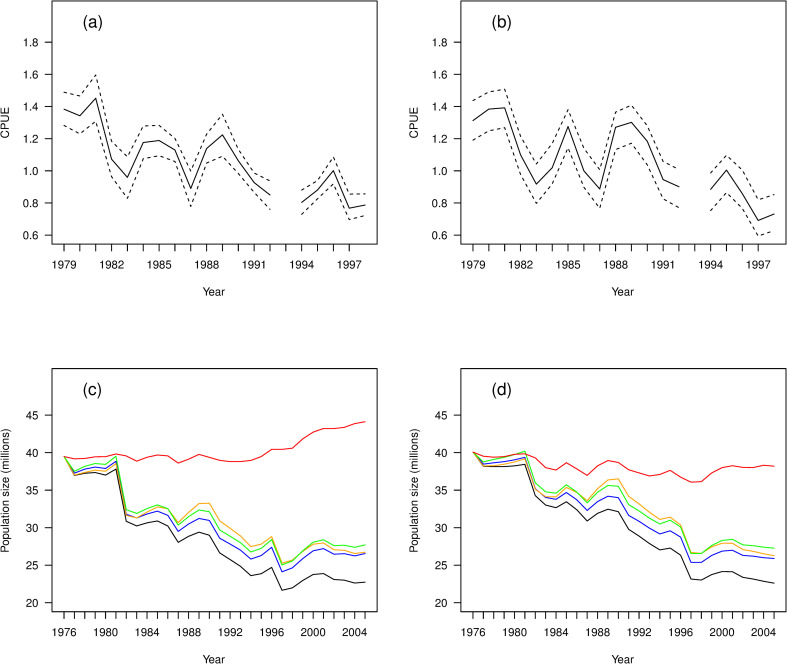
Effects of including process error in the best-fitting model on the estimation of CPUE and the effects of climate, harvesting, bycatch, and weka-depredation. Posterior means (solid) and 95% credible intervals (dashed) for a) CPUE from the best model (S–1F0) and b) CPUE from the modified best model, plus posterior means of population size (black), predicted population size in the absence of harvest (blue), the absence of bycatch (orange), the absence of weka (green), and the absence of a relationship between SOI and adult survival (red) c) from the best model (S–1F0) and d) from the modified best model.

Using the values *σ*_*s*_ = 0.37 and *σ*_*f*_ = 0.72 in the modified model, we obtained the results shown in Figs 5 and 6. The relationship between SOI and adult survival was slightly weaker in the modified model, with the posterior means at the extremes being 0.84 and 0.95, compared to 0.78 and 0.96 for the original model; the uncertainty was also generally greater for the modified model, as we would expect, with the mean increase in the width of the 95% credible interval being 33% (Fig 5A vs 5B). This difference was generally less marked for fecundity, with the mean increase in the width being 6% (Fig 5C vs 5D). For the modified model the posterior probability that *β*_*s*_ > 0 was 0.73, compared to 0.97 for the original best model; the posterior probability that *β*_*f*_ > 0 was 0.99, identical to that for the original model. Qualitatively, these results are what we would expect, with addition of process errors leading to greater uncertainty about the association between SOI and both adult survival and fecundity. Having said that, the modified model still provides evidence that strong El Niño events are associated with decreases in adult survival and fecundity, while strong La Niña events are associated with increases in these parameters. The process errors in the modified model correspond to non-SOI factors that might affect the temporal variation in adult survival and fecundity. As discussed earlier, the data do not allow estimation of the true size of these error terms, but the sensitivity analysis indicates that even if these errors are large the conclusions concerning the potential impact of SOI are qualitatively unchanged.

The posterior prediction intervals for CPUE obtained from the modified model were generally wider than hose for the original model, again as we would expect, the mean increase in the width of the interval being 34% (Fig 6A vs 6B). The posterior means showing the estimated effects of climate, harvest, bycatch, and weka depredation during the period 1976–2005 indicate a weaker effect of SOI, as we would expect from the above results (Fig 5A vs 5B). The posterior mean for population size obtained from the modified model shows a similar pattern of decline to that from the original model (Fig 6C vs 6D; black lines). For the modified model, the posterior mean for the population decline is 1.9% per year, the same (to 1dp) as that obtained from the original model; the 95% credible interval for this decline is 1.4% to 2.5%, which is 39% wider than the corresponding interval from the original model, as we would expect.

The impacts of harvest, bycatch, and depredation by weka are again estimated to be similar (Fig 6D; blue, orange and green lines respectively), with the posterior means for the annual decline being 1.5% (0.7–2.2%), 1.4% (0.7–2.1%) or 1.3% (0.6–2.0%) for a population not exposed to harvest, bycatch or weka, respectively. These estimates are almost identical to those from the original model, but the 95% credible intervals are 43%, 55% and 37% wider respectively, again as we would expect. The impact of the relationship between SOI and adult survival is still stronger than that from any of the other threats, but to a lesser extent than for the original model (Fig 6C vs 6D; red lines); the posterior mean for the growth rate suggests an annual decline of 0.5% in the absence of this relationship, compared to a growth of 0.2% for the original model. The 95% credible interval from the modified model lay between an annual decline of 2.4% and an annual increase of 3.1%, which is 27% wider than the interval for the original model, which lay between an annual decline of 2.0% and an annual increase of 2.4%. The impact of the relationship between SOI and fecundity is not shown in Fig 6C and 6D, as the effect was again negligible, for both the modified and the original model.

Identical comparisons for the modified and original versions of the other models in Table 3 led to similar conclusions to those discussed here. Note that we have not computed model-averaged posterior means and credible intervals for the modified versions of the models in Table 3, as we could not estimate the process-error standard deviations, which makes comparison, and hence averaging, of the modified models impossible. As we deliberately chose high values for *σ*_*s*_ and *σ*_*f*_, we would expect that adding the true process errors to the best model would lead to qualitatively similar, but less marked, differences between the original and modified versions of the model, compared to those evident in Figs 5 and 6.

**Fig 7:**
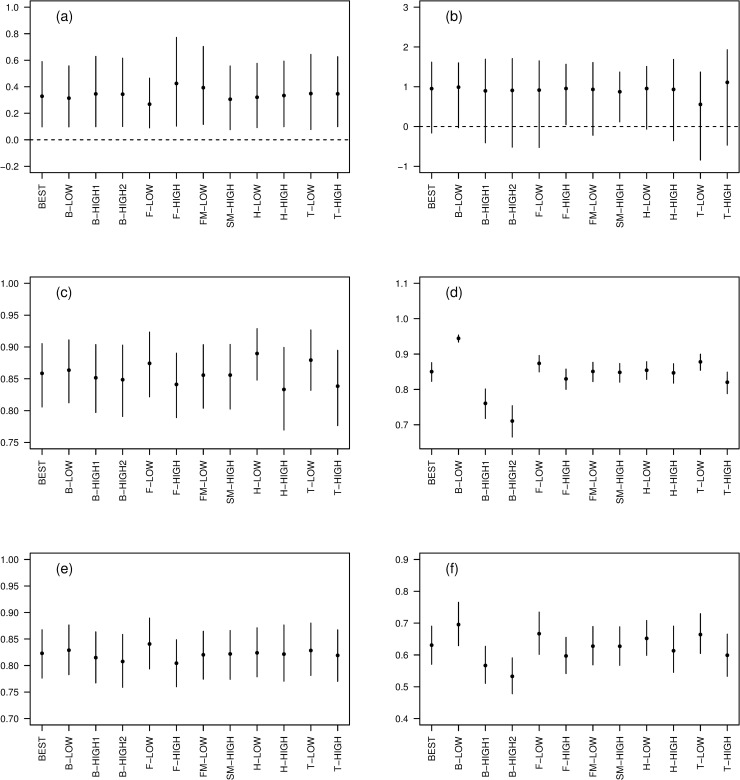
Sensitivity analysis for the best-fitting demographic model. Comparison of estimates, between models with different input parameter values, of a) the coefficient of the relationship between fecundity and SOI, b) the coefficient of the relationship between adult survival and SOI, c) the predicted population size in 2005 relative to the predicted population size in 2005 in the absence of harvesting, d) the predicted population size in 2005 relative to the predicted population size in 2005 in the absence of fisheries bycatch, e) the predicted population size in 2005 relative to the predicted population size in 2005 in the absence of weka depredation, and f) the predicted population size in 2005 relative to the predicted population size in 2005 in the absence of harvesting, bycatch and weka depredation. Dots are posterior means and error bars show the central 95% credible intervals.

Our second sensitivity analysis did not identify any substantial changes in model output when the models in Table 2 were fitted and compared to the best model (Fig 7). Posterior distributions of *β*_*f*_ were similar across all models, with zero outside all the 95% credible intervals (Fig 7A). All but two of the alternative models (F-HIGH and SM-HIGH) had a lower 95% credible limit for *β*_*s*_ which was below zero (Fig 7B), but the posterior probabilities of being less than zero were relatively small, ranging from 0.02 to 0.15. Inferences about the relative impacts of harvesting were similar across all models (Fig 7C), the largest differences being between low and high estimates of harvest (H-LOW vs H-HIGH), and between low and high estimates of trend (T-LOW vs T-HIGH). For bycatch, there were predictable differences between some of the models (Fig 7D); for example, use of high estimates of bycatch (B-HIGH1 and B-HIGH2) led to a larger estimate of the effect of bycatch on the predicted population size in 2005, and vice versa for low estimates of bycatch (B-LOW). For weka depredation, there was little variation between the models (Fig 7E). Finally, for all three sources of mortality combined (Fig 7F), the largest differences were between the models with different estimates of bycatch (B-LOW vs B-HIGH1 and B-HIGH2).

## Discussion

### Climate induced variation in abundance

Climate is increasingly being recognised as an important driver of seabird dynamics, with a number of studies detecting relationships between abundance or demographic parameters and large-scale climate indices or physical variables that are themselves correlated with climate [60, 61]. This was also the case for our analysis, where incorporation of climate-related variation in vital rates explained much of the variation in the data. In general, our models support the work of [8] and in addition we have extended their work by focusing on relationships between climate indices and individual demographic rates, allowing climate to separately influence survival and fecundity. Interestingly, the optimal lags for the two relationships differed, and non-negligible weight was given to models with different lags for adult survival. The potential link between tītī demography and El Niño and La Niña events that we identify here has also been detected in tropical pelagic seabirds elsewhere in the Pacific [62]. Tītī exhibit very complex spatial dynamics, which includes migration to the Northern Pacific during the Austral winter and foraging trips over vast areas of the Southern Ocean during the breeding season [2]. The different relationships between SOI and demographic rates likely reflect this complexity and are presumably related to the physical (e.g. wind) and/or biological (prey abundance and quality) mechanisms influencing the different rates in different areas and times.

A significant hurdle to assessing the influence of climate on population dynamics is the need to reduce the candidate set of climate covariates to a practical number [63]. While relationships with more specific environmental variables could be investigated, our understanding of the mechanisms influencing the response of the population to climate is poor, and thus modelling large-scale climatic events would appear to be a suitable first approach. Furthermore, large-scale climate indices can in some cases be better indicators of the climatic events that affect populations [64] due to the difficulties in identifying the specific climate variables responsible, or the possibility that they are multivariate. There remains the possibility that other indices may have been suitable for inclusion in the modelling, for instance, those calculated from conditions in the North Pacific, where tītī spend the Austral winter. Future investigation of relationships with conditions during this stage of the life-cycle would certainly be interesting and could potentially allow the roles of the different regions in regulating shearwater numbers to be assessed. The sensitivity analysis in which we added process errors to the best model suggests that we can be confident that there is a relationship between SOI and the vital rates, even after allowing for other (climate) factors that might influence these rates.

### Relative impacts of other mortality sources

Despite climate-driven variation in abundance, the functional form of the relationships between climate indices and demographic rates, and the distribution pattern of climate index values, some of the reduction in abundance over the period 1976–2005 is accounted for by non-climate factors. It appears that declines can largely be explained by the synergistic effects of fisheries bycatch, depredation by introduced animals, human harvesting of chicks and, for certain periods of time, unfavourable climatic conditions. Perhaps the most important finding of this study is that none of the individual threats to the tītī population appear to be capable of explaining the observed dynamics alone. However, the limited role of climate in the decline over 1976–2005 is partly the result of the series of favourable SOI values in the very late 1990s and early 2000s, that appeared to allow some rebuilding of the population. Just prior to this, in 1997, the "pristine" modelled population was predicted to be at its lowest point, a decline of 17.6% relative to 1976, which can presumably be attributed mainly to the prevailing climatic conditions at that time, given that the other main mortality sources had been “factored out”. This demonstrates the potential influence that climate can have, and suggests that future abundance of tītī may well depend on the frequency and severity of climate fluctuations, providing a strong relationship between demography and climate persists. While these inferences rely on accurate measurement of the magnitude of each threat, our sensitivity analyses indicate that these results are relatively robust to a wide range of potential model misspecifications.

We model non-natural mortality additively under the assumption that estimation of the vital rates is independent of the mortality sources. For instance, estimates of adult survival are made for the period after the banning of driftnet bycatch, and fecundity is estimated in the absence of predation and harvesting. There remains the possibility that modelled sources of mortality may also have contributed to population growth rates, the most obvious of which is the possible reduction of tītī prey through intense commercial fishing pressure, as has been proposed by [65] as a potential agent in the declines of other top predators in the Southern Ocean. Some unmodelled mortality is to be expected and will be included in the error terms when the model is fitted to the data, thereby limiting its effect on inferences. Substantial modelled mortality is more serious, but our results suggest this has not occurred. Firstly, vital rates presumably include most other mortality sources if they occurred over the period the rate was estimated. For instance, the period of intense depletion of many fish species in the Southern Ocean occurred prior to, or during, the early part of the model period [65]; if this suppressed tītī vital rates we would expect it to be accounted for in our parameter estimates e.g. mortality of adults would be included in the "natural" mortality estimate. If a modelled mortality source was occurring and not accounted for by the vital rates then we would expect either the modelled mortality to be unable to reproduce the observed decline or significant shifts in the posterior densities of vital rates would have occurred during model fitting to accommodate the mismatch. Neither of these phenomena were observed.

### Limitations in the modelling of tītī dynamics

Robust modelling of animal population dynamics relies on a number of conditions being met including; the use of a model that adequately approximates the true dynamics of the system, a dataset that provides sufficient information to estimate all parameters, and accommodation of all relevant sources of uncertainty. While model diagnostics and sensitivity analyses can be useful in checking some aspects of model development and fit, there will generally be a number of caveats required when the results are reported. Two processes that were not included in our modelling and sensitivity analyses were the potential for island-specific vital rates (including immigration between islands) and density dependence.

Lack of data on vital rates and on the magnitude of different mortality sources, at an island-scale over the study period, precludes construction of spatially explicit models. Therefore, if our region-wide approach is to be robust, mortality rates among islands must either be similar, or immigration rates between island populations must be sufficient to ameliorate site-specific differences in mortality. It seems reasonable to assume that both bycatch and climate influences are likely to be similar across the different island populations, given the time and spatial scales at which these processes operate. In contrast, harvesting rates of chicks display moderate variation between islands [19], and introduced predators are present on only some islands [13]. A valuable future research topic would be to determine the level of mixing between populations that would be necessary to offset any differences in population growth rate that resulted from population-specific vital rates. For now we assume that our model provides a fair reflection of the overall New Zealand population, given that the demographic parameters to which growth rate is most sensitive (adult and juvenile survival) are presumably similar across populations. However, there remains the possibility that changes in abundance in some individual populations with very different mortality rates (e.g. very high harvest rate of chicks) will be misrepresented by our model. While potentially important to the viability of an individual colony, this issue is unlikely to affect conclusions for the entire regional population [1, 19]. Furthermore, immigration between island populations are increasingly being detected in a range of long-lived seabird species [66, 67]. Although these rates have yet to be estimated for tītī, if similar levels occur we can expect the results of our modelling to be robust to the assumption that the spatial scale of our model is adequate.

An important consideration when developing the structure of population models is whether to accommodate density dependence, and if so, how it should be parametrised. We assume that density dependence is not operating over the density range encountered in the study period. While the presence and strength of density dependence can, in principle, be estimated from time-series data, reliable estimation is difficult to achieve, even for simple models (e.g. [68, 69]). Robust inferences generally rely on abundance datasets spanning a wide range of densities, from near to well below carrying capacity [70]. Tītī, like other long-lived species are expected to exhibit a population growth response curve [71] that decreases in a convex manner as density increases [72]. This suggests that density dependent changes in vital rates should primarily occur over the density range close to carrying capacity. Declines in tītī populations are believed to have occurred from well before our study period [9], so we expect population density to be well below the range at which significant density dependent changes in vital rates occur.

In spite of the caveats given above, the purpose of constructing our models was to make inferences about the main influences on the dynamics of the New Zealand tītī population, utilizing models that capture the most important properties of the system. In this respect our research appears to have been successful, given that the best models account for much of the variation in the dataset, and this is supported by the consistency of our results across the sensitivity analysis.

### Management implications

Our primary aim in this paper has been to gain some understanding of the relative contributions of climate, bycatch, harvest and depredation by weka to the decline of the New Zealand tītī population over the period 1976–2005. Population projections based on our results will be the focus of future research, in which we plan to compare different management strategies, using both simulation and sensitivities obtained from a deterministic version of the model [34]. The results presented here already suggest that climate change, particularly the frequency and intensity of future El Niño and La Niña events, will be an important factor in determining the sustainability of the population.

Some past threats to the population have been alleviated but others remain and will be difficult to mitigate. Fisheries bycatch levels have declined dramatically since peak mortality prior to the international ban on driftnet fishing. Bycatch is no longer thought to be an important issue for tītī, unlike other seabird species; while it persists [43], mortality rates would appear to have little impact at the population level, unless there are very large unreported sources of death as a consequence of illegal large-scale fisheries [73]. There are additionally few actions that can be taken by harvest managers to curb the impacts of El Niño-Southern Oscillation (ENSO), with most benefits for tītī persistence probably relying on international climate change policy. An improved understanding of how predicted climate changes will influence ENSO patterns will be a key to future research. Case studies of demographic relationships with climate change, such as our one, are important for policy makers to grasp the consequences of their actions.

Depredation of chicks by introduced predators, and harvesting by humans, can be more readily addressed in management strategies than can the impacts of climate. However, our results suggest that removing these factors has fewer benefits for the tītī population than alleviating climate drivers. Easing harvesting pressure could help by increasing the rate of recovery, especially after El Niño knock-down events. A similar outcome would be envisaged if the depredation effects of weka on some islands were removed. Preliminary versions of our model have been utilised in the past to guide operations aimed at eradicating introduced predators (i.e. ship rats) from tītī breeding islands [74]. In that case management aimed to mitigate the effects of an oil spill in the North Pacific which affected migrating tītī. The models we have presented here will be a useful tool for prioritizing which islands should be the focus of future eradication or control programs, and the likely conservation gains that would result. It is envisioned that the model presented will continue to form the basis for quantitative assessments of competing management strategies.

## Supporting information

S1 TableValues of $z_{t}^{f}$ and $z_{t}^{s}$.Annual measures of the Southern Oscillation Index used to model the relationship between climate and both fecundity ($z_{t}^{f}$) and adult survival ($z_{t}^{s}$).(DOCX)
