# Supplementary material for: Separating the effects of climate, bycatch, predation and harvesting on tītī (Ardenna grisea) population dynamics in New Zealand: A model-based assessment
Source: PLoS One. 2020 Dec 14;15(12):e0243794. doi: 10.1371/journal.pone.0243794 (PMC7735597; doi:10.1371/journal.pone.0243794)
Supplement: S1 Table — Annual measures of the Southern Oscillation Index used to model the relationship between climate and both fecundity (ztf) and adult survival (zts). (DOCX) [file pone.0243794.s001.docx]

**S1 Table. Values of** $\boldsymbol{z}_{\boldsymbol{t}}^{\boldsymbol{f}}$**and** $\boldsymbol{z}_{\boldsymbol{t}}^{\boldsymbol{s}}$**.** Annual measures of the Southern Oscillation Index used to model the relationship between climate and both fecundity ($z_{t}^{f}$) and adult survival ($z_{t}^{s}$).

| Year (*t*) | $z_{t}^{f}$ | $z_{t}^{s}$ |
| --- | --- | --- |
|  |  |  |
|  |  |  |
| 1974 | 1.00 |  |
| 1975 | 0.49 | 1.58 |
| 1976 | -0.51 | -0.20 |
| 1977 | -0.31 | -1.10 |
| 1978 | -0.08 | 0.18 |
| 1979 | -0.29 | -0.15 |
| 1980 | 0.17 | -0.32 |
| 1981 | -0.62 | 0.45 |
| 1982 | -1.32 | -2.03 |
| 1983 | 0.06 | 0.01 |
| 1984 | 0.17 | 0.06 |
| 1985 | -0.04 | 0.09 |
| 1986 | -1.27 | -0.44 |
| 1987 | 0.37 | -0.94 |
| 1988 | 1.04 | 1.08 |
| 1989 | -0.09 | 0.25 |
| 1990 | -0.54 | 0.00 |
| 1991 | -0.99 | -1.21 |
| 1992 | -1.00 | -0.65 |
| 1993 | -0.91 | -0.76 |
| 1994 | -0.36 | -1.02 |
| 1995 | 0.46 | 0.01 |
| 1996 | -0.63 | 0.56 |
| 1997 | -0.61 | -1.67 |
| 1998 | 0.79 | 0.79 |
| 1999 | 0.75 | 0.77 |
| 2000 | 0.42 | 0.76 |
| 2001 | -0.38 | -0.10 |
| 2002 | -0.47 | -0.69 |
| 2003 | -0.21 | -0.12 |
| 2004 | -0.52 | -0.61 |
| 2005 | 0.04 | 0.13 |
| 2006 |  | -0.47 |
